# Impact of chromium histidinate on high fat diet induced obesity in rats

**DOI:** 10.1186/1743-7075-8-28

**Published:** 2011-05-03

**Authors:** Mehmet Tuzcu, Nurhan Sahin, Cemal Orhan, Can Ali Agca, Fatih Akdemir, Zeynep Tuzcu, James Komorowski, Kazim Sahin

**Affiliations:** 1 Department of Biology, Faculty of Science Firat University, 23119 Elazig, Turkey; 2 Department of Animal Nutrition, Faculty of Veterinary Science, Firat University, 23119 Elazig, Turkey; 3 Department of Animal Nutrition, Faculty of Veterinary Science, Dicle University, 23119 Diyarbakir, Turkey; 4 Nutrition 21 Inc, 4 Manhattanville Road, Purchase, NY, 10577, USA

## Abstract

**Background:**

Chromium (Cr) is an essential trace element that has garnered interest for use as a weight loss aid, but its molecular mechanism in obesity is not clear. In this study, an attempt has been made to investigate the effects of chromium histidinate (CrHis) on glucose transporter-2 (GLUT-2), nuclear factor erythroid 2-related factor 2 (Nrf2), heme oxygenase-1 (HO-1), nuclear factor-kappa B (NF-κB p65) and the oxidative stress marker 4-hydroxynonenal adducts (HNE) expressions in liver of rats fed high fat diet (HFD).

**Methods:**

Male Wistar rats (n = 40, 8 wk-old) were divided into four groups. Group I was fed a standard diet (12% of calories as fat); Group II was fed a standard diet and supplemented with 110 μg CrHis/kg BW/d; Group III was fed a HFD (40% of calories as fat); Group IV was fed HFD and supplemented with 110 μg CrHis/kg BW/d.

**Results:**

Rats fed HFD possessed greater serum insulin (40 *vs*.33 pmol/L) and glucose (158 *vs*. 143 mg/dL) concentration and less liver Cr (44 *vs*.82 μg/g) concentration than rats fed the control diet. However, rats supplemented with CrHis had greater liver Cr and serum insulin and lower glucose concentration in rats fed HFD (*P *< 0.05). The hepatic nuclear factor-kappa B (NF-κB p65) and HNE were increased in high fat group compared to control group, but reduced by the CrHis administration (*P *< 0.05). The levels of hepatic Nrf2 and HO-1 were increased by supplementation of CrHis (*P *< 0.05).

**Conclusion:**

These findings demonstrate that supplementation of CrHis is protective against obesity, at least in part, through Nrf2-mediated induction of HO-1 in rats fed high fat diet.

## Background

The global prevalence of overweight and obesity is increasing rapidly worldwide among adults as well as among children and adolescents in places where high dietary fat intake and this is leading to dramatic increases in complications such as hyperlipidemia [1], fatty liver [2], type II diabetes mellitus [3] and cardiovascular diseases [4]. High-fat diet (HFD) is associated with alterations in liver chemistry and structure, increased risk of liver damage ranging from steatosis to steatohepatitis, cirrhosis, and even hepatocellular degeneration. High dietary fat intake is considered to be an important factor in the development of insulin resistance [5-7], oxidative stress [8], inflammation, abnormal mitochondria, and increased collagen content [9,10]. Elevated levels of oxidative stress can potentially impair cellular glucose metabolism via a variety of mechanisms, including redox imbalance and insulin resistance.

Several pharmacological agents such as insulin-sensitizing agents may be used to reduce or control the body weight and obesity. One of such agents, chromium (Cr) has been examined in some animal studies and clinical studies for its anti-obesity effects [11]. Cr is essential for the maintenance of normal metabolism of carbohydrate and lipids [12]. Inadequate amounts of Cr may result in improper functioning of the metabolic process and lead to a number of physiological disorders that increase risk for diabetes and cardiovascular diseases including elevated circulating insulin, glucose, triglycerides, total cholesterol, reduced HDL-cholesterol and impaired immune function [12,13]. Laboratory and clinical evidence indicate that chromium supplementation may improve insulin sensitivity by enhancing intracellular signaling [14,15]. Cr complexes have also been shown to reduce oxidative stress in diabetic rats [16,17]. Chromium histidinate (CrHis) is a stable compound with a better absorption than other forms of chromium compounds tested in human subjects [18]. Anderson *et al. *[18] observed that men and women absorbed an average 3.1 mcg of Cr from the CrHis complex, compared to 1.8 mcg from chromium picolinate (CrPic), 0.4 mcg from chromium chloride and 0.2 mcg from chromium polynicotinate. Cr may attenuate some effects of a HFD, mainly body fat accretion [19].

Nuclear factor-kappa B (NF-κB p65) and nuclear factor-E2-related factor 2 (Nrf2) have been drawn an increasing attention for their role in protecting tissue injury such as liver disorders [20]. Nuclear factor-kappa B, crucial for the priming phase of liver regeneration, has been shown to be involved in promoting hepatocyte proliferation and inhibiting apoptosis of liver cells [20,21]. Nuclear factor-E2-related factor 2 is a transcription factor that is activated by oxidative stress and electrophiles that regulates the expression of numerous detoxifying and antioxidant genes. Studies have shown that Nrf2 protects the liver from xenobiotic toxicity [22,23], and it was reported that HFD reduced the mRNA expression of Nrf2 and its target genes in mice [23]. The synthetic oleanolic triterpenoid 1-[2-cyano-3,12-dioxooleana-1,9 (11)-dien-28-oyl]imidazole (an extremely potent activator of Nrf2 signaling) effectively prevents increase in the body weight following a high-fat diet, showing that Nrf2 can be an attractive target for management of obesogenesis [22]. However, the effects of Cr supplementation on liver NF-κB, Nrf2 and HO-1 activation in rats fed a HFD are not known. In this study, it has been investigated the effects of a HFD on glucose metabolism, liver damage and oxidative stress, and to further examine whether this damage could be repaired by CrHis. Accordingly, the effects of CrHis on liver tissue were determined in the context of indices of oxidative stress (HNE) and inflammation (e.g., hepatic expression of NF-κB).

## Methods

### Animals and diets

Male Wistar rats (n = 40, 8 weeks old) weighing 200-215 g were purchased from Firat University Laboratory Animal Research Center (Elazig, Turkey). The animals were housed at the temperature of 22 ± 2°C, humidity of 55 ± 5%, and with a 12/12 h light/dark cycle throughout the experiment. The experiment was conducted under the protocol approved by the Firat University. All procedures involving rats were conducted in strict compliance with relevant laws, the Animal Welfare Act, Public Health Services Policy, and guidelines established by the Institutional Animal Care and Use Committee of the university. Rats were fed standard diet (12% of calories as fat) or high fat diet (HFD, 40% of calories as fat). During the animal experimentation, rats in the treatment groups were supplemented with CrHis (Nutrition 21, NY, USA) via drinking water. Ingredients and chemical composition of the basal (control) diet are shown in Table 1. The diets were stored at 4°C cold chamber.

**Table 1:** Ingredient and nutrient composition of the diets

Ingredients (g/kg)	Normal Diet	High-Fat Diet (HFD)
Casein	200.0	200.0
Starch	615.0	145.0
Sucrose	-	150.0
Corn oil	80.0	-
Beef tallow	-	400.0
Cellulose	50.0	50.0
Vitamin-Mineral Premix^1^	50.0	50.0
DL-Methionine	3.0	3.0
Choline chloride	2.0	2.0
Chromium, mg/kg	0.066	0.097

^1^The vitamin-mineral premix provides the following (per kg): all-*trans*-retinyl acetate, 1.8 mg; cholecalciferol, 0.025 mg; all-*rac*-a-tocopherol acetate, 12,5 mg; menadione (menadione sodium bisulfate), 1.1 mg; riboflavin, 4.4 mg; thiamine (thiamine mononitrate), 1.1 mg; vitamin B-6, 2.2 mg; niacin, 35 mg; Ca-pantothenate, 10 mg; vitamin B-12, 0.02 mg; folic acid, 0.55 mg; *d*-biotin, 0.1 mg. manganese (from manganese oxide), 40 mg; iron (from iron sulfate), 12.5 mg; zinc (from zinc oxide), 25 mg; copper (from copper sulfate), 3.5 mg; iodine (from potassium iodide), 0.3 mg; selenium (from sodium selenite), 0.15 mg; choline chloride, 175 mg.

### Experimental Design

After 1 week of adaptation period, the rats were randomly divided into four groups as (i) control group: rats were fed a standard diet (12% of calories as fat); (ii) CrHis group: rats were fed the standard diet and received CrHis; (iii) HFD group: rats were fed a high-fat diet (40% of calories as fat); (iv) HFD+CrHis group: rats were fed a high-fat diet (40% of calories as fat) and received CrHis. CrHis (Nutrition 21, Inc., Purchase, NY, USA) was dissolved in water and administered at a concentration of 110 μg/kg.d in the drinking water containing 5.53 μg Cr/L for 12 weeks to get 8 μg Cr/day which is an equivalent dose of 560 μg Cr for a 70 kg adult human [17].

### Laboratory Analyses

For the measurement of biochemical markers, blood samples were collected from the tail vein of each rat prior to scarification by cervical dislocation. Blood samples were centrifuged at 3000 *g *for 10 min and sera were separated. Fasting glucose concentrations were determined at euthanasia, using the glucose-oxidase method and reading in a glucometer (Accu-chek, Roche Diagnostic, Germany). Serum insulin (Linco Research Inc, St. Charles, MO, USA) concentration was measured by ELISA (EL_x_-800, Bio-Tek Instruments Inc, City, VT, USA).

After digesting with a mixture of concentrated HNO_3 _(65% Merck, Darmstadt, Germany) and H_2_O_2 _(30% Merck) in a Microwave Digestion System (Berghoff, Eningen, Germany), liver samples and blood sera were analyzed for Cr content using graphite furnace atomic absorption spectrophotometer (AAnalyst 800, Perkin-Elmer Corp., Norwalk, CT, USA) as described by Dogukan et al. [24].

### Western blot analyses

Liver samples were also analyzed for the expression of GLUT-2, NF-κB, Nrf2, HO-1 and HNE using the western blot technique. In all groups, the liver was removed from sacrificed rats. Small pieces of the liver samples in each group of animals were pooled together for Western blot analysis. Protein extraction was performed as follows: The sample was homogenized in 1 ml ice-cold of hypotonic buffer A [10 mM HEPES (pH 7.8), 10 mM KCl, 2 mM MgCl2, 1 mM DTT, 0.1 mM EDTA, 0.1 mM phenylmethylsulfonyl-fluoride (PMSF)]. 80 μl of 10% Nonidet P-40 (NP-40) solution was added to the homogenates, and the mixture was then centrifuged for 2 min at 14,000 g. The supernatant was collected as a cytosolic fraction for the analysis of GLUT-2, HO-1 and HNE. The precipitated nuclei were washed once with 500 μl of buffer A plus 40 μl of 10% NP-40, centrifuged, resuspended in 200 μl of buffer C [50 mM HEPES (pH 7.8), 50 mM KCl, 300 mM NaCl, 0.1 mM EDTA, 1 mM DTT, 0.1 mM PMSF, 20% glycerol] and centrifuged for 5 min at 14,800 g. The supernatant containing nuclear proteins was collected for the analysis of Nrf2 and NFkB p65 [25]. Concentration of the protein was determined according to the procedure described by Lowry *et al. *[26] using a protein assay kit supplied by Sigma, St. Louis, MO, USA. Sodium dodecyl sulfate-polyacrylamide gel electrophoresis sample buffer containing 2% *β*-mercaptoethanol was added to the supernatant. Equal amounts of protein (50 μg) were electrophoresed and subsequently transferred to nitrocellulose membranes (Schleicher and Schuell Inc., Keene, NH, USA). Nitrocellulose blots were washed twice for 5 min each in PBS and blocked with 1% bovine serum albumin in PBS for 1 h prior to application of the primary antibody. The antibody against Nrf2 and HNE was purchased from Santa Cruz Biotechnology, Inc. (Santa Cruz, CA, USA). Antibody against GLUT-2, NF-κB and HO-1 was purchased from Abcam (Cambridge, UK). Primary antibody was diluted (1:1000) in the same buffer containing 0.05% Tween-20. The nitrocellulose membrane was incubated overnight at 4°C with protein antibody. The blots were washed and incubated with horseradish peroxidase-conjugated goat anti-mouse IgG (Abcam, Cambridge, UK). Specific binding was detected using diaminobenzidine and H_2_O_2 _as substrates. Protein loading was controlled using a monoclonal mouse antibody against β-actin antibody (A5316; Sigma). Blots were performed at least three times to confirm the reproducibility of the results. Bands were analyzed densitometrically using an image analysis system (Image J; National Institute of Health, Bethesda, USA).

### Statistical analysis

The data were analyzed using the General Linear Model (GLM) procedure of SAS software [27]. Significant differences at 5% among treatment means were determined using Fisher's post hoc test for all groups.

## Results

### Glucose, Insulin and Cr Levels

As shown in Table 2 the measurement of the total body weight revealed that rats fed HFD were heavier than the control rats (317 *vs*. 262 g). In addition, rats fed HFD had higher serum insulin (40 *vs*. 33 pmol/L) and glucose concentration (158 *vs*. 143 mg/dL), and less liver Cr concentration (44 *vs*. 82 μg/g) than rats fed a control diet (Table 2) (*P *< 0.05). CrHis administration in the control group did not affect body weight, insulin and glucose levels in the serum but did increase Cr concentration in the liver. CrHis administration significantly reduced the total body weight and glucose level in rats fed HFD (Table 2). However, HFD rats supplemented with CrHis had higher liver Cr and serum insulin than rats fed HFD (*P *< 0.05) (Table 2).

**Table 2:** The effect of CrHis supplementation on levels of glucose, insulin and liver Cr in rats fed the high-fat diet (n = 10)

Items	Groups			
	
	Control	CrHis	HFD	HFD+ CrHis
Body mass, g	265 ± 6.5 ^c^	262 ± 5.8 ^c^	317 ± 5.3 ^a^	293 ± 4.2^b^
Insulin, pmol/L	33.5 ± 0.3^c^	33.9 ± 0.1^c^	39.3 ± 0.2^a^	40.6 ± 0.2^b^
Glucose, mg/dl	125 ± 5.1^c^	122 ± 6.9^c^	158 ± 4.6^a^	143 ± 5.2^b^
Liver Cr, μg/g	55.4 ± 3.8^c^	82.3 ± 2.2 ^a^	44.6 ± 4.6^d^	61.3 ± 3.1 ^b^

a-c: Means in the same line without a common superscript differ significantly (*P *< 0.05).

HFD: High fat diet

CrHis: Chromium histidinate

### Western Blots Analyses of GLUT-2, Nrf2, HO-1, NF-κB p65 and HNE

**Figure 1:**
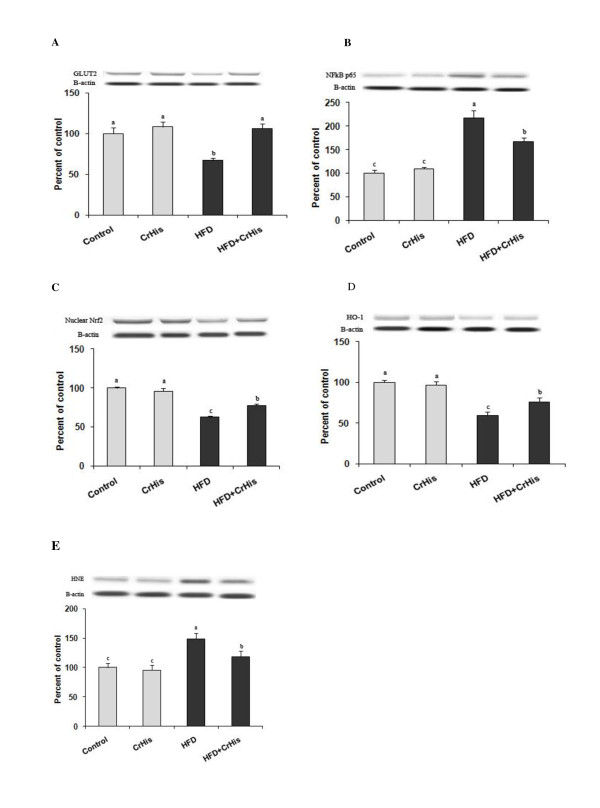
**Effect of HFD and CrHis on liver GLUT-2 (A), NFκB (B), Nrf2 (C), HO-1 (D) and HNE (E) expressions in rats fed high fat diet**. The intensity of the bands was quantified by densitometric analysis and normalized with corresponding β-actin. Values are means ± standard error of the mean. Western blot analysis was repeated at least 3 times (n = 3) and a representative blot is shown. Data points with different superscripts are significantly different at the level of p < 0.05 by Fisher's multiple comparison test.

Figure 1 shows the effects of CrHis on GLUT-2, Nrf2, HO-1 and NF-κB expression in the liver of rats by western blot. Based on band densities, liver GLUT-2 expression was significantly decreased in rats fed HFD diets compared to the control rats (Figure 1A). However, protein expression of GLUT-2 in the liver was increased to the normal level in the rats fed HFD by CrHis supplemention (Figure 1A). The expression of NF-κB in liver showed that high dietary fat intake increased the expression of NF-κB compared to the control (*P *< 0.05). However, the increased NF-κB expression in liver after HFD treatment was decreased by CrHis treatment (*P *< 0.05) (Figure 1B). The accumulation of Nrf2 in the nuclear fraction and HO-1 was significantly lower in liver of HFD-treated rats than that of the control rats (*P *< 0.05) (Figure 1C and 1D). In the group of rats fed HFD+CrHis, however, Nrf2 accumulation in the nuclear fraction of liver was significantly increased as compared to HFD treatment alone (*P *< 0.05), but it was lower than that of seen in the control group (Figure 1C). HO-1 expression of the HFD-induced liver injury group was statistically lower than control (P < 0.05), whereas there was a significant increase in the level of HO-1 expression between the HFD-induced liver injury group and the HFD+CrHis-treated group (P < 0.05) (Figure 1D). The analysis of expression of HNE by western blot in liver showed that high dietary fat intake increased the protein expression of HNE compared to the levels seen in the control (*P *< 0.05) (Figure 1E). However, the increased HNE expression in liver after HFD treatment was decreased by CrHis treatment (*P *< 0.05) (Figure 1E).

## Discussion

Insulin resistance has been shown to be the major contributing factor to the metabolic syndrome, which comprises a cluster of risk factors for conditions such as obesity, dyslipidemia, hypertension, and hyperglycemia [10,28]. Previous studies showed that a high fat diet can lead to visceral obesity in rodent animal models [29-31]. Dietary trivalent Cr has been shown to play an important role in metabolic disorders associated with insulin resistance and hyperglycemia by providing significant beneficial effects in the insulin system [32]. Cr acts by interfering with the number of receptors accessible to insulin, modulation of their affinity to the hormone, and the disruption of intracellular signal transduction [15,33,34]. Cr supplementation has been also shown to inhibit increase in inflammatory markers and oxidative stress levels in cultured monocytes exposed to high glucose levels [17,35,36]. The purpose of the current study was to examine whether CrHis could play a role in the prevention of liver damage of rats fed a HFD.

It has been shown that a HFD results in significant increase in body weight, blood glucose and insulin levels [37,38]. In the present study, it has been observed that CrHis supplementation reduced serum levels of glucose and increased serum insulin and liver Cr levels in rats fed HFD (Table 2). The mechanisms of Cr action on insulin signaling pathways have been extensively studied over recent years. These effects appear to be related to an increase in insulin sensitivity, with the mechanisms explained recently in great detail by Vincent and Bennett [39]. Chromium is thought to increase both the binding of insulin to its cell surface receptors, and the number of insulin receptors [14]. Similar results demonstrating the ability of chromium to improve glucose and insulin tolerance and to reduce blood glucose and lipids have been previously reported [17,40-42]. Daily supplementation of trivalent Cr-containing milk powder reduced serum levels of glucose, insulin and triglycerides, and improved glucose and insulin tolerance [43].

The present study has shown that CrHis supplementation induced GLUT-2 activity, increased Nrf2 and HO-1 expression and downregulated NF-κB and HNE activity in liver of rats fed by HFD (Figure 1). Glucose transporter-2 is the major glucose transporter protein involved in transport of glucose across hepatocyte, either from inside or outside depending upon the insulin stimuli. There was no previous study investigating the effects of Cr supplementation on the GLUT-2 in liver with which to compare this study. However, CrPic was reported to enhance skeletal muscle glucose transporter-4 (GLUT-4) translocation and insulin sensitivity in a rat model of obesity and insulin resistance [44] and also recruits intracellular localization of GLUT-4 to the cytoplasmic side of the plasma membrane of murine adipocytes [45]. In a similar experiment, improvement in glucose tolerance was attributed to enhanced membrane-associated GLUT-4 in obese rats after insulin stimulation [44]. Cr supplementation activated postreceptor insulin signaling such as increasing insulin resistance-associated (IRS1) and GLUT-4 expression, stimulating phosphoinositide 3-kinases (PI3-K) and Akt activity, downregulating c-Jun N-terminal kinases (JNK) activity and decreasing IRS1 phosphorylation in skeletal muscles [43].

It has been reported that high dietary fat intake promoted inflammation and NF-κB activation [46]. Nuclear factor-kappa B in most cells is bound with an inhibitory protein of nuclear factor-κB (IκB) to form a latent, inactive transcription factor in the cytoplasm. In the case of oxidative stress and various cytokines, NF-κB is released rapidly from IκB to activate the gene expression of several cytokines, chemotactic and matrix proteins involved in inflammation, immunological responses and/or proliferation [8,34]. The results have shown that consumption of a HFD enhanced NF-κB p65 subunit activation in rat liver, which supports our observation of high fat diet-induced oxidative stress. Fan *et al. *[47] reported that NF-κB binding activity was higher in the rats fed HFD diet than that in the controls. However, the effect of CrHis on the NF-κB pathway is still unclear. Similarly, Cr dinicocysteinate -treated rats showed decreased levels of activated NF-κB when compared to diabetic rats [34].

Recent research has identified Nrf2 as a key transcription factor for combating hepatic oxidative stress [48]. Nuclear factor erythroid 2-related factor 2 controls the antioxidant response element (ARE)-dependent gene regulation in response to oxidative stress. Nrf2 sequestered in the cytoplasm by the cytosolic repressor Kelch-like ECH-associated protein 1 (Keap 1) plays an important role in the maintenance of the cellular redox balance. Keap1 links Nrf2 to the cytoskeleton to retain Nrf2 in the cytoplasm, thereby promoting its degradation. Oxidative stress facilitates Nrf2 to escape Keap1-mediated proteasomal degradation, leading to Nrf2 stabilization, subsequent nuclear translocation, and binding to ARE [49]. Nrf2 induces expression of antioxidant enzymes and phase II detoxifying proteins, such as heme oxygenase-1 by binding to antioxidant responsive elements in the promoters of these genes [48]. However, it is not known whether CrHis plays a role in the Nrf2/HO-1 pathway in liver. The present study demonstrates for the first time that supplementing CrHis increases expression of Nrf2 and HO-1 in liver of rats fed HFD (Figure 1). This finding suggests that Cr may be involved in stabilization and activation of Nrf2. However, Nrf2 plays a protective role against liver injury by regulating expression of antioxidant genes and phase II drug-metabolizing enzymes in liver [50]. On the other hand, Nrf2 inhibits lipid accumulation and oxidative stress in mouse liver after feeding a HFD, probably by interfering with lipogenic and cholesterologenic pathways [23].

Several studies have shown elevated oxidative stress markers in obesity [51-53]. High intake of dietary fat enhances ROS overproduction which increases lipid peroxidation [46]. Increased body fat stimulates excessive reactive oxygen species production by NADPH oxidase activation [54]. The reaction of free radicals with membrane lipids causes the formation of lipid peroxidation products including several aldehydic compounds, one of which is highly toxic and called 4-hydroxynonenal (HNE). 4-hydroxynonenal is frequently measured as indicators of lipid peroxidation and oxidative stress *in vivo *and is considered as an index of oxidative stress. In the present study, expression of HNE in liver of rats fed HFD decreased when dietary CrHis was supplemented. CrHis supplementation did not alter these parameters in control rats. Significantly lower expression of HNE was observed in animals receiving CrHis supplementation. These findings indicate significant positive associations between CrHis intake and HNE expression for rats fed high fat diets. Cr, an insulin cofactor, is postulated to function to augment antioxidant defense system [17,36]. Similar results have been reported by Preuss *et al. *[16] who have explained a decrease in hepatic TBARS formation by supplementation of CrPic and chromium nicotinate in rats. Similarly, Cr dinicocysteinate significantly reduced lipid peroxidation and increased blood vitamin C and adiponectin levels in ZDF rats [34].

## Conclusion

The present investigation has shown that high dietary fat intake can induce changes in metabolic profile such as increased body weight, serum insulin and glucose concentrations, and decreased liver chromium concentrations and glucose transporter-2, Nrf2, HO-1 expressions and increased NF-κB and HNE expressions. These adverse alterations could be partially reversed by the supplemental CrHis. These results suggest that supplementation of CrHis would be effective on protection of obesity through Nrf2-mediated induction of heme oxygenase-1 in subjects when fed with HFD.

## List of Abbreviations

ARE: antioxidant response element; CrHis: chromium histidinate; GLUT: glucose transporter; GTF: glucose tolerance factor; HDL: High density lipoprotein; HFD: high fat diet; HNE: 4-hydroxynonenal adducts; HO-1: heme oxygenase-1; IRS1: insulin resistance-associated; IκB: nuclear factor-B; JNK: c-Jun N-terminal kinases; Keap 1:Kelch-like ECH-associated protein 1; NF-κB p65: nuclear factor-kappa B; Nrf2: Nuclear factor erythroid 2-related factor 2; PI3-K: phosphoinositide 3-kinases.

## Competing interests

The study was funded by Nutrition 21, Inc., NY, USA. Nutrition 21 alsosupplied the chromium histidinate used in the study. James Komorowskiis an employee of Nutrition 21, the distributors of chromium histidinateunder a license from the USDA.

## Authors' contributions

MT participated in study design, data collection, laboratory analyses, wrote the first draft of the manuscript, and presented his poster at the Seventh International Nutrition and Dietetics Congress in Istanbul, Turkey, 2010. NS participated in study design, laboratory analyses and data interpretation and wrote the first draft of the manuscript. CO, CAA, FA and ZT participated in data collection and laboratory analyses and assisted in every aspect of the study. JK participated in study design, interpretation and preparation of the manuscript. KS participated in organization of the study and data interpretation and preparation of the manuscript. All authors read and approved the final manuscript.
